# Effect of Sulfonation Degree and PVDF Content on the Structure and Transport Properties of SPEEK/PVDF Blend Membranes

**DOI:** 10.3390/polym11040676

**Published:** 2019-04-13

**Authors:** Shaojian He, Shaoxiong Zhai, Chong Zhang, Yang Xue, Wei Yang, Jun Lin

**Affiliations:** 1 Beijing Key Laboratory of Energy Safety and Clean Use, North China Electric Power University, Beijing 102206, China; heshaojian@ncepu.edu.cn (S.H.); ncepuzhaisx@163.com (S.Z.); 2 State Key Laboratory of Advanced Power Transmission Technology, Global Energy interconnection Research Institute, Beijing 102211, China; 18611602136@163.com (C.Z.); 13810231165@163.com (W.Y.); 3 State Key Laboratory of Multi-phase Complex System, Institute of Process Engineering, Chinese Academy of Science, Beijing 100190, China

**Keywords:** sulfonated poly (ether ether ketone), polyvinylidene fluoride, blend membrane, sulfonation degree, transport properties

## Abstract

Sulfonated poly (ether ether ketone) (SPEEK) with four different sulfonation degrees (SDs) were prepared, and mixed with polyvinylidene fluoride (PVDF) to prepare four series of SPEEK/PVDF blend membranes. The miscibility between SPEEK and PVDF was investigated by observing the micro-morphologies. The miscible blend membranes were found in the SPEEK/PVDF blend membranes in which either SPEEK had relatively low SD or consisted of low content of one component (either SPEEK or PVDF). The PVDF crystallinity was found to decrease in the SPEEK/PVDF membranes that had better blend miscibility. With the increase of PVDF content, all the blend membranes exhibited the decreased proton conductivity and methanol permeability, and the miscible blend membranes decreased more slowly than the immiscible ones.

## 1. Introduction

Perfluorosulfonic acid (PFSA) membranes, such as Nafion, have been commonly considered as the proton exchange membranes (PEMs) for fuel cells due to their high proton conductivity and excellent chemical and mechanical properties [1,2,3,4]. However, these materials are expensive and have relatively poor resistance to methanol transport [5,6,7,8]. Studies have shown that PEMs based on sulfonated aromatic hydrocarbon polymers exhibit proton conductivities comparable to Nafion and could become the alternatives to Nafion [9,10,11,12]. Among them, sulfonated poly (ether ether ketone) (SPEEK) has great potential due to its excellent mechanical properties, good chemical resistance, and low cost [13,14]. Unfortunately, large swelling in methanol remains a problem for SPEEK membranes.

Polymer blending is a widely used approach for obtaining materials with specifically targeted properties [15], and each component of the blend can be chosen to meet specific requirements. PVDF is considered to be the structural component by virtue of its excellent thermal stability and mechanical strength [16]. Although the incorporation of nonconductive material could usually cause a reduction in the proton conductivity of the membrane [16,17], the hydrophobic nature of PVDF can help increase the resistance to methanol crossover. Therefore, SPEEK/PVDF blends were investigated mainly for the applications in direct methanol fuel cells (DMFCs) [18,19,20,21,22], where the influence of blend miscibility on the transport properties of polymer blend membranes becomes particularly important. Inan et al. [23] studied the SPEEK/PVDF blends in which SPEEK had the sulfonation degree (SD) of 70%. They found that there was depression of melting temperature for PVDF. Since no phase separation was observed in their membranes, they suggested that PVDF and SPEEK be mixed with high compatibility. Such a conclusion was supported by Xue et al.’s work [24], in which the SD of SPEEK was 78%, very close to Inan et al.’s work. However, when using SPEEK with a SD of 47%, Jung et al. [18] showed that SPEEK/PVDF blend was not sufficiently compatible when PVDF was more than 10 wt.%, and good compatibility was achieved only when PVDF content was no more than 5 wt.%. Ren et al. [19] found that all of the blend membranes (with SPEEK/PVDF weight proportion of 9/1, 8/2 and 5/5) had smooth and homogeneous surfaces, but there was no mention of the SD of SPEEK. Wootthikanokkhan et al. [20] observed the SPEEK/PVDF blends (the SD of SPEEK was 78%) with 30 wt.% and 10 wt.% PVDF had higher water uptake than pure SPEEK, which was attributed to the immiscibility between SPEEK and PVDF.

In this work, SPEEK with different SDs were prepared, each of which was mixed with PVDF with different contents, and solution cast to prepare SPEEK/PVDF blend membranes. The structure and transport properties of the blend membranes were investigated. Through this work, we aimed to investigate the influence of blend miscibility on the transport properties of the blend membranes composed of hydrophilic SPEEK and hydrophobic PVDF. Knowledge of the conductivity–miscibility relationships should be useful for designing ionomer blends for DMFC.

## 2. Experimental

### 2.1. Materials

Poly (ether ether ketone) (PEEK) (VICTREX 150PF) was purchased from Victrex (Lancashire, UK). PVDF (M_w_ = 530,000) was supplied by Fluka. Sulfuric acid (analytical grade from Merck, Darmstadt, Germany), methanol (analytical grade), and dimethylacetamide (DMAc, analytical grade) were used as received.

### 2.2. Preparation of SPEEK and SPEEK/PVDF Blend Membranes

Sulfonation of PEEK was carried out using concentrated sulfuric acid according to the following procedure: 15 g dried PEEK was entirely dissolved in 300 mL concentrated sulfuric acid (98%) by stirring at 800 rpm at room temperature for a certain period of time before the solution was dumped into the ice/water mixture to precipitate the sulfonate polymer (SPEEK), which were repeatedly washed with deionized (DI) water until the pH reach 7. Then SPEEK was dried in the convection oven at 80 °C for 24 h. By using the titration method according to the literature [13,25], the ion exchange capacities (IECs) and SDs of the prepared SPEEK were determined, and the results are listed in Table 1.

SPEEK and PVDF solution was prepared separately in DMAc with the concentration of 10 w/v %. A certain amount of the PVDF solution was added to the SPEEK solution and stirred at room temperature for 24 h. Then the mixture was cast onto a flat glass substrate. After being dried at 80 °C for 24 h and then at 120 °C under vacuum for another 12 h, the SPEEK/PVDF blend membranes were peeled from the glass surface. Before tests, the blend membranes were kept in 1.0 M sulfuric acid solution for 24 h and then washed with deionized water to remove any residual acid. The membrane samples are labeled as SPEEKx/PVDF-y, where x% refers to the SD of SPEEK and y% refers to the weight percentage of PVDF in the blend membranes.

### 2.3. Measurements

Optical micrographs were obtained from a metallographic microscope (10XB-PC, Shanghai Yongheng Optical Instrument Manufacturing Co. Ltd., Shanghai, China). The morphologies of the blend membranes were observed by a scanning electron microscopy (SEM) (SU8010, Hitachi Co. Ltd., Tokyo, Japan) at an accelerating voltage of 5 kV. Differential scanning calorimetry (DSC) experiments were performed in a dynamic mode using STA 499 F3 DSC (NETZSCH, Selb, Germany). The samples were heated from 40 °C to 230 °C and then back to 40 °C at 20 °C/min, and finally from 40 °C to 230 °C at 10 °C/min.

Before water uptake measurement, the membranes were soaked in DI water at room temperature for 24 h. After being wiped off the water on the membrane surface, the wet membranes were weighed immediately. The water uptake was calculated by the following equation:

(1) $$\mathrm{water}\;\mathrm{uptake}=\frac{m_{\mathrm{wet}}-m_{\mathrm{dry}}}{m_{\mathrm{dry}}}\times 100\%$$

where *m*_wet_ and *m*_dry_ are the weight of the wet and dry membranes, respectively.

AC impedance spectroscopy of the wet membranes was recorded by an electrochemical workstation (Zennium Pro., Zahner, Kronach, Germany), in which the membrane samples were fixed by a BekkTech four-probe conductivity cell in liquid water. The proton conductivity (*σ*) was calculated using the following equation:

(2) $$\sigma =\frac{l}{R\cdot A}$$

where *l* is the distance between the two inner electrodes, *A* is the cross-section area of the membranes, and *R* is the membrane resistance.

Methanol permeability was measured at 40 °C in a two-chamber diffusion cell, where one chamber was filled with 1.0 M methanol solution and the other (receiving) chamber was filled with DI water. The vertically positioned membrane sample was sandwiched between these two chambers. The methanol concentration in the receiving chamber was determined using gas chromatography technique with a flame ionization detector (FID). The methanol permeability (*P*) was calculated by the following equation:

(3) $$P=\frac{V_{B}L(dc_{t}/dt)}{c_{0}A}$$

where *c*_0_ is the initial methanol concentration in the chamber filled with methanol, *t* is the permeation time, *c_t_* is the methanol concentration in the receiving chamber, *A* is the exposed area of the membrane, *L* is the membrane thickness, and *V*_B_ is the volume of the methanol solution.

All the measurements were conducted at 25 °C unless mentioned otherwise.

## 3. Results and Discussion

### 3.1. Microstructure of Blend Membranes

**Figure 1:**
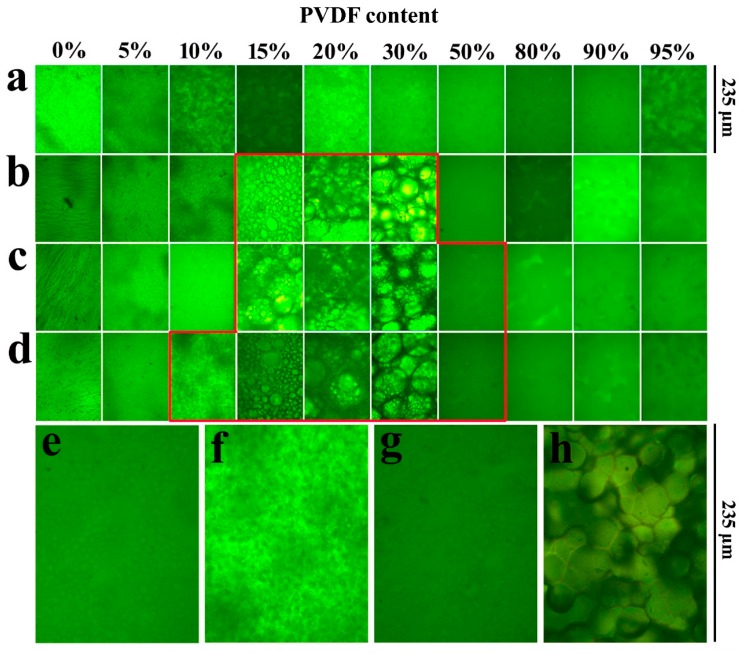
Optical micrographs of (**a**) SPEEK44/PVDF, (**b**) SPEEK56/PVDF, (**c**) SPEEK67/PVDF and (**d**) SPEEK73/PVDF blend membranes with different PVDF contents, (**e**) SPEEK67/PVDF-50, (**f**) SPEEK73/PVDF-10, (**g**) SPEEK73/PVDF-50 blend membranes and (**h**) PVDF.

The miscibility behavior of the SPEEK/PVDF blends is studied by optical microscopy. As shown in Figure 1, the pure SPEEK membranes with different SDs show almost the same morphologies, and the pure PVDF membrane exhibits crystal morphology (Figure 1h). The SPEEK44/PVDF blend membranes show no sign of phase separation over the whole range of PVDF content as shown in Figure 1a. At a slightly higher SD for SPEEK, evidence of an immiscibility gap appears. As shown in Figure 1b, the PVDF crystalline domain could be observed for the SPEEK56/PVDF blend membranes with the PVDF content ranging between 15% and 30%, indicating the immiscibility between SPEEK and PVDF. Moreover, the signs of phase separation appear within the PVDF content of 15%~50% and 10%~50% for the SPEEK67/PVDF and SPEEK73/PVDF blend membranes shown in Figure 1c,d, respectively. The SPEEK with lower SD is more hydrophobic due to the existence of less sulfonate groups. Because of the hydrophobic nature of PVDF, the blend membranes with SPEEK of lower SD show the stronger intermolecular interaction between the two components (SPEEK and PVDF) than those with SPEEK of higher SD, resulting in the better miscibility. Optical micrographs of the SPEEK/PVDF blend membranes demonstrate that the SPEEK of lower SD is more compatible with PVDF than that of higher SD, and SPEEK and PVDF are miscible when a small amount of PVDF is introduced, or the amount of PVDF is more than that of SPEEK in the blend membranes.

**Figure 2:**
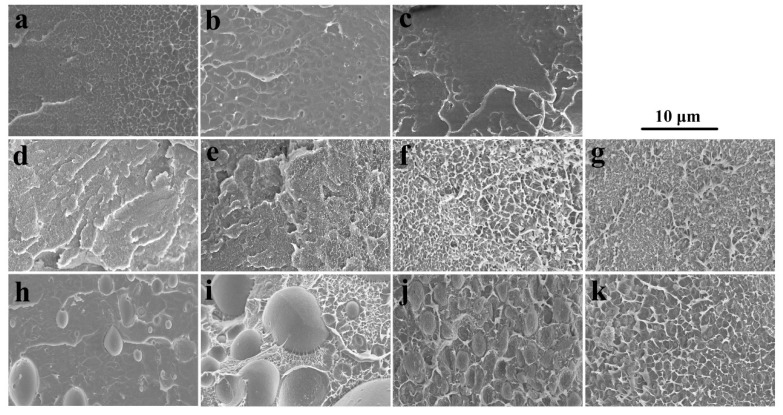
SEM images of (**a**) SPEEK44, (**b**) SPEEK73, (**c**) PVDF, (**d**) SPEEK44/PVDF-10, (**e**) SPEEK44/PVDF-30, (**f**) SPEEK44/PVDF-50, (**g**) SPEEK44/PVDF-80, (**h**) SPEEK73/PVDF-10, (**i**) SPEEK73/PVDF-30, (**j**) SPEEK73/PVDF-50 and (**k**) SPEEK73/PVDF-80 blend membranes.

**Figure 3:**
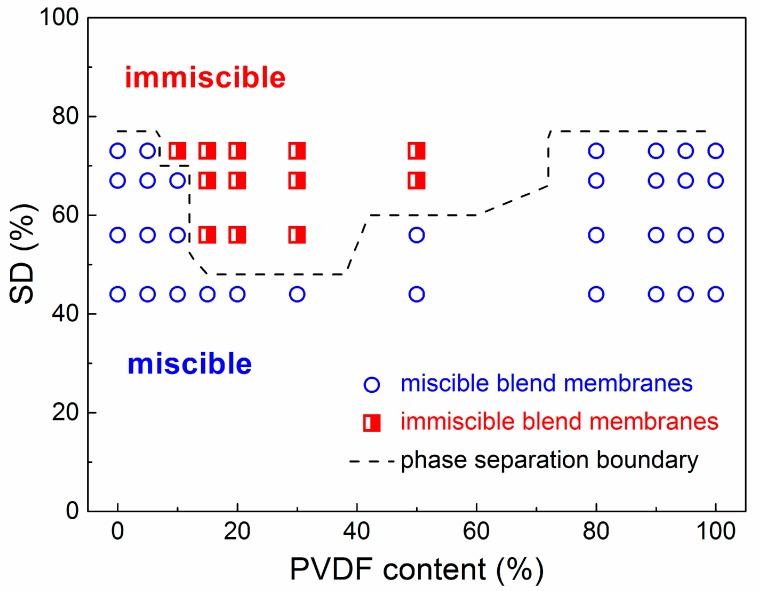
Isothermal phase diagram of miscible and immiscible SPEEK/PVDF blend membranes at different SDs versus PVDF content.

SEM images of SPEEK44/PVDF and SPEEK73/PVDF blend membranes with different PVDF contents are compared in Figure 2 to further illustrate the phase separation. The SPEEK44/PVDF blend membranes exhibit the homogeneous structure and no evidence of phase separation is observed in the fracture surface (Figure 2d–g). As for the SPEEK73/PVDF blend membranes, it is found that some ‘islands’ (PVDF) with the size of 2~5 μm are dispersed in the ‘sea’ (SPEEK) for the blend membrane with 10% PVDF (Figure 2h), and the size of the ‘islands’ increases to 8~10 μm when the PVDF content increases to 30% (Figure 2i). With the PVDF content being increased further to 50%, the size of the ‘islands’ decreases down to 3~4 μm, while the number of the ‘islands’ increases (Figure 2j). These ‘islands’ turn to be more and more continuous. However, when the PVDF content rises to 80%, all the ‘islands’ disappear and the morphology becomes homogeneous, indicating no phase separation for the SPEEK73/PVDF-80 blend membrane (Figure 2k). Both SEM images and optical micrographs demonstrate that the SPEEK44 is miscible with PVDF, and the phase separation occurs in the SPEEK/PVDF blend membranes, in which the SPEEK has the SD higher than 44%. As shown in Figure 3, an isothermal phase diagram is used to summarize the findings of phase separation from the optical microscopy and SEM analysis of the SPEEK/PVDF blend membranes to distinguish the miscibility between SPEEK and PVDF.

### 3.2. DSC Analysis

**Figure 4:**
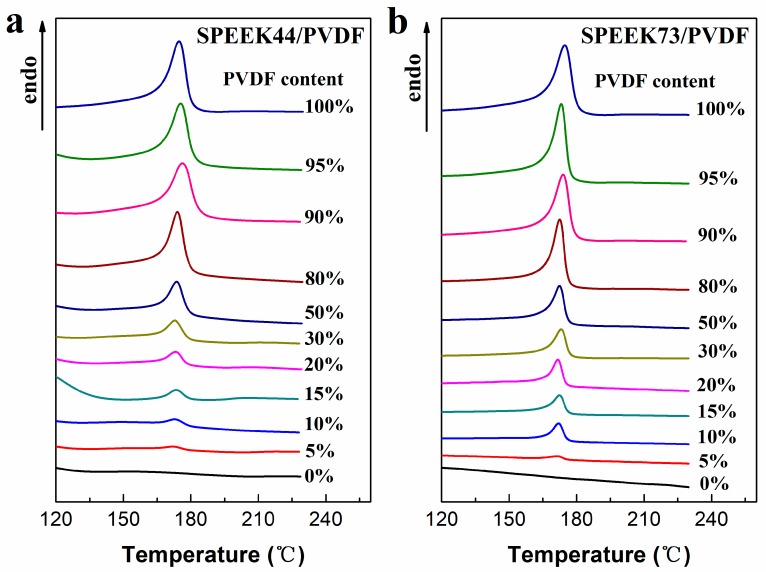
DSC curves of (**a**) SPEEK44/PVDF and (**b**) SPEEK73/PVDF blend membranes with different PVDF contents.

The DSC curves of SPEEK/PVDF blend membranes are shown in Figure 4. The melting point, melting enthalpy and crystallinity of the blend membranes are listed in Table 2, in which the crystallinity of PVDF in the blend membranes was calculated using the following equation:

(4) $$PVDF\;crystallinity=\frac{\Delta H_{f}}{\Delta H_{f}^{*}\cdot \omega_{PVDF}}\times 100\%$$

where Δ*H_f_* is the melting enthalpy of PVDF derived from the area of endothermic peak in DSC curves and the heating rate (peak area/heating rate), ΔH_f_^*^ is the melting enthalpy of the perfect PVDF crystal (104.5 J·g^−1^) [26], and *ω_PVDF_* is the weight content of PVDF in the blend membranes. For the melting points corresponding to the maximum endothermic temperature, there exists no obvious trend in either SPEEK44/PVDF or SPEEK73/PVDF blend membranes with different PVDF contents. As for the PVDF crystallinity in the SPEEK/PVDF blend membranes, there exists a dependence on the PVDF content and the SD of SPEEK. When the PVDF content is less than 50%, both SPEEK44/PVDF and SPEEK73/PVDF blend membranes show the increased PVDF crystallinity with the increasing PVDF content, and, the SPEEK73/PVDF blend membranes exhibit a higher PVDF crystallinity than the SPEEK44/PVDF blend membranes with the same PVDF content. Nevertheless, when PVDF content is more than 50%, PVDF crystallinity levels off at around 40% that equals to the crystallinity of the pristine PVDF membrane. Generally, the good compatibility between two polymers should result in the reduced crystallinity. Therefore, the results mentioned above indicate that the SPEEK of lower SD is more compatible with PVDF than that of higher SD, especially at low PVDF content. When the PVDF content is no less than a specific content (80% for the SPEEK44/PVDF blend membrane and 50% for the SPEEK73/PVDF blend membrane), the PVDF component in the blend membranes completely transforms from ‘island’ to ‘sea’. Therefore, the effect of the other component (SPEEK) on the PVDF crystallinity should be weakened, resulting in the similar crystallinity in the blend membranes to that in the pristine PVDF.

### 3.3. Proton Conductivity

**Figure 5:**
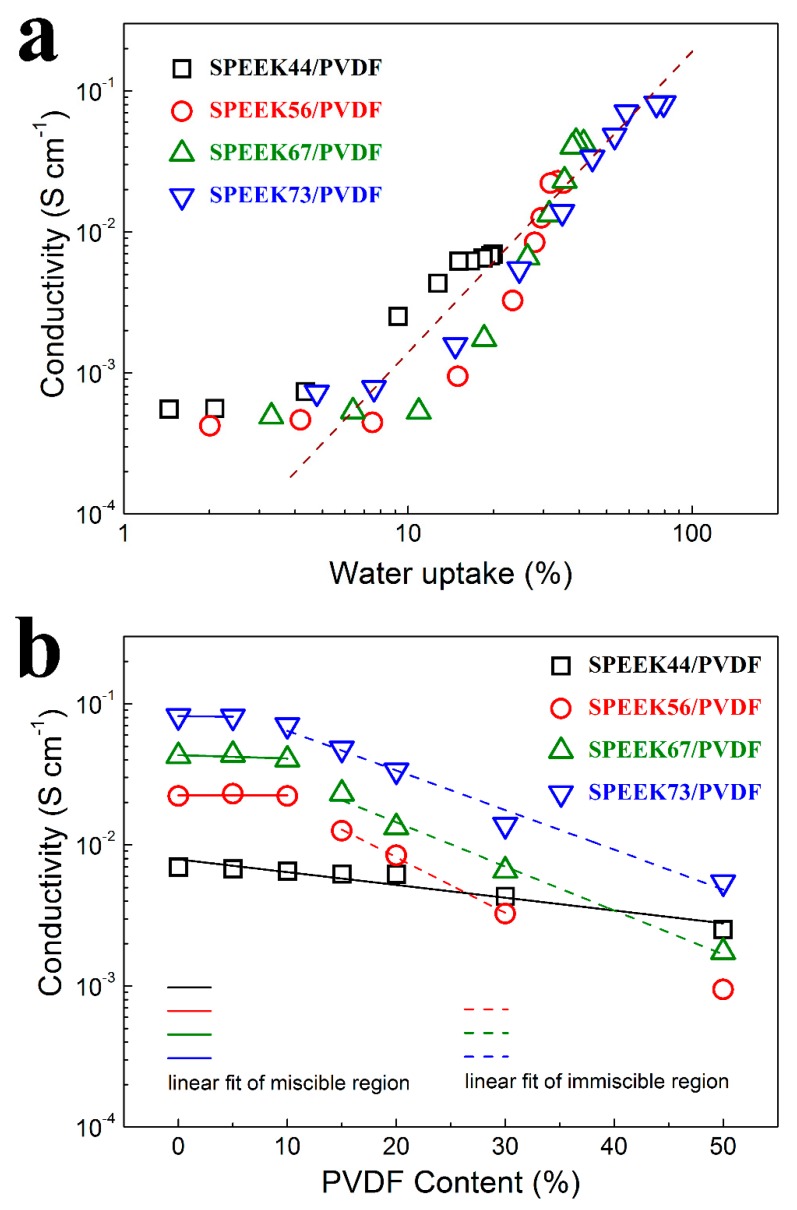
Proton conductivity as a function of (**a**) water uptake and (**b**) PVDF content for SPEEK/PVDF blend membranes.

For the SPEEK/PVDF blend membranes with different PVDF contents, the proton conductivities are plotted as a function of water uptake as shown in Figure 5a. When the water uptake is more than 10%, the proton conductivity of all these membranes starts to increase significantly, which is commonly observed for the PEMs reported in the literature [27,28]. Among these membranes, the SPEEK44/PVDF blend membranes exhibit much higher proton conductivity than the other blend membranes at the water uptake between 10~20%, which may result from the fact that these SPEEK44/PVDF membranes have larger concentration of protons. Such results also indicate that the better miscibility in the SPEEK44/PVDF blend membranes could help reduce the water uptake and provide more continuous proton pathways.

The proton conductivity of the SPEEK/PVDF blend membranes as a function of the PVDF content is shown in Figure 5b. For the miscible SPEEK44/PVDF blend membranes with different PVDF contents, there is a nearly linear decrease in logarithmic proton conductivity (lg*σ*) with increasing PVDF content. For the miscible SPEEK73/PVDF blend membranes with PVDF content lower than 10%, there is also a decrease in lg*σ* with increasing PVDF content. However, for the immiscible SPEEK73/PVDF blend membranes with PVDF content between 10% and 50%, lg*σ* drops abruptly (almost linearly) with the increasing PVDF content. Moreover, with further increase of the PVDF content, the decrease in lg*σ* turns slow again for the SPEEK73/PVDF blend membranes that are miscible. The similar phenomena are also found for both SPEEK56/PVDF and SPEEK67/PVDF blend membranes. The results indicate that the miscible blend membranes exhibit a slower decrease in lg*σ* than the immiscible blend membranes with the increasing content of ionic insulator (PVDF), which can be explained by comparing the morphologies of the miscible and immiscible blend membranes. For the immiscible blend membranes, the phase separation is observed, where the PVDF ‘islands’ are embedded in the continuous SPEEK ‘sea’ as illustrated in Figure 2h–j. The existence of ion-insulating PVDF ‘islands’ should not only reduce the content of ion-conductor but also create more tortuous proton transport pathways, thus leading to the severe deterioration in lgσ for the blend membranes. As for the miscible blend membranes, in which SPEEK and PVDF well compatible with each other forming bi-continuous structure as illustrated in Figure 2d–g,k, the decline in lgσ should be only attributed to the reduction of the ion-conductor content. Therefore, the slower decline in lg*σ* is expected.

### 3.4. Methanol Permeability

**Figure 6:**
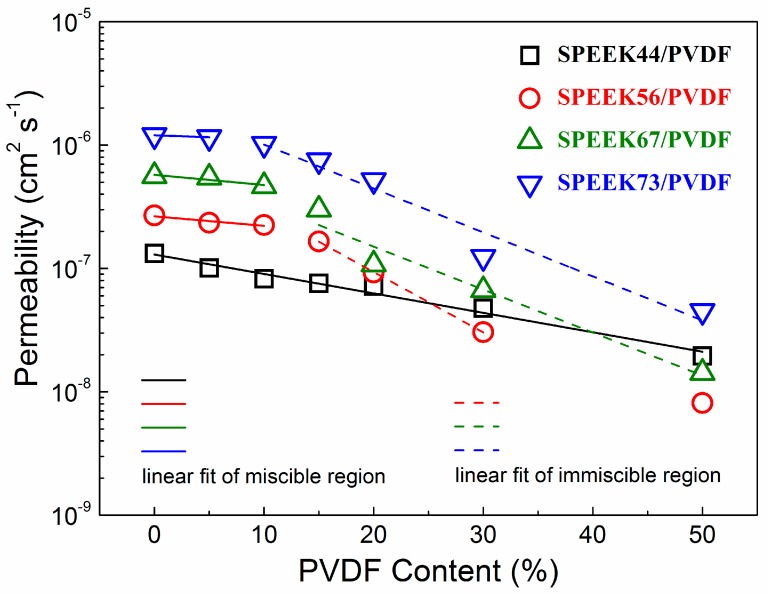
Methanol permeability as a function of PVDF content for SPEEK/PVDF blend membranes.

The methanol permeability as a function of the PVDF content for the SPEEK/PVDF blend membranes is shown in Figure 6. Similar to the behavior of the membrane proton conductivity, the methanol permeability decreases with the increasing PVDF content. The decrease in logarithmic methanol permeability (lg*P*) of the miscible SPEEK/PVDF blend membranes is much slower than that of the immiscible blend membranes. The ionic insulating PVDF also acts as the barrier to the transport of methanol, thus causing methanol permeability to exhibit similar behavior as the proton conductivity. For the miscible blend membranes, as far as the methanol barrier effect is concerned, the PVDF content rather than the crystallinity play a significant role in reducing the methanol permeation: the SPEEK44/PVDF with 15% PVDF has almost the same crystallinity as the SPEEK73/PVDF with 5% PVDF; however, the decrease of methanol permeability is much more for the SPEEK44/PVDF with 15% PVDF. Such behavior can be understood due to the fact that PVDF is hydrophobic and impede the movement of methanol, regardless of its crystallinity.

Regarding proton conductivity results shown in Figure 5b, we can see that the decrease of methanol permeability for the miscible blend is a little faster than that of proton conductivity, indicating that the selectivity (the ratio of proton conductivity to methanol permeability) would increase with the PVDF content. Therefore, taking into account that the miscible SPEEK/PVDF blend exhibits higher proton conductivity with the same water uptake as shown in Figure 5a, we expect that a miscible polymer blend could achieve high performance in DMFCs when the content of non-ionic conductive component reaches close to the miscible-immiscible boundary.

## 4. Conclusions

In this work, four series of SPEEK/PVDF blend membranes, which included SPEEK of four different, were prepared to investigate the influence of miscibility on the transport properties of blend membranes. The SPEEK of the lower SD was more compatible with PVDF than that of higher SD. SPEEK and PVDF were compatible with each other when a low content of PVDF was introduced or the PVDF content was more than the SPEEK content in the blend membranes. The better miscibility between SPEEK and PVDF can result in the lower PVDF crystallinity in the blend membranes. For any series of SPEEK/PVDF blend membranes, both proton conductivity and methanol permeability decreased with the increasing PVDF content. The decrease in the proton conductivity and methanol permeability of the miscible SPEEK/PVDF blend membranes was much slower than that of the immiscible blend membranes with the same PVDF content. Therefore, it is expected that that a miscible polymer blend could achieve high performance in DMFCs when the content of non-ionic conductive component reaches close to the miscible-immiscible boundary.

## Figures and Tables

**Table 1:** IECs and SDs of the prepared SPEEK.

Sulfonation Time (h)	IEC (mmol g^−1^)	SD (%)
12	1.33	44
17	1.64	56
36	1.89	67
48	2.01	73

**Table 2:** Melting point, melting enthalpy and crystallinity of PVDF in the SPEEK44/PVDF and SPEEK73/PVDF blend membranes derived from DSC curves.

PVDF Content (%)	SPEEK44/PVDF			SPEEK73/PVDF		
	Melting Point (°C)	Melting Enthalpy (J·g^−1^)	PVDF Crystallinity (%)	Melting Point (°C)	Melting Enthalpy (J·g^−1^)	PVDF Crystallinity (%)
0	-	0	0	-	0	0
5	172.0	0.38	7	171.4	0.91	17
10	172.8	1.27	12	172.0	2.00	19
15	173.5	2.54	16	172.4	3.87	25
20	173.2	4.16	20	171.7	6.10	29
30	172.9	8.72	29	173.2	11.9	38
50	173.7	17.0	33	172.4	21.8	41
80	174.1	34.9	42	172.5	36.1	43
90	176.3	42.3	45	174.0	41.2	44
95	175.5	43.1	44	173.2	43.0	43
100	174.8	47.8	46	174.8	47.8	46
